# Centralising a loss of consciousness to the central medial thalamus

**DOI:** 10.1177/23982128241306549

**Published:** 2024-12-16

**Authors:** Florence Rawlings-Mortimer, Jeffrey W. Dalley

**Affiliations:** 1 Department of Psychology, University of Cambridge, Cambridge, UK; 2 Department of Psychiatry, Herchel Smith Building for Brain and Mind Sciences, University of Cambridge, Cambridge, UK

**Keywords:** Consciousness, thalamus, GABA

## Abstract

Although a role of the thalamus in different arousal and awareness states is well established, there is a surprising lack of knowledge on subregional specificity within this complex, multinucleated structure of the diencephalon. In their recent paper ‘Extrasynaptic GABA-A receptors in central medial thalamus mediate anaesthesia in rats’, Muheyati et al. evaluated whether GABA_A_ receptors expressed in the central medial (CM), paraventricular (PV) or lateral mediodorsal (MD) nuclei of the thalamus contribute to the loss of the righting reflex (LORR) in rats. Deficits in this reflex have previously been interpreted as a surrogate marker of altered levels of consciousness. Using a range of convergent techniques, the authors report the novel finding that delta subunit-expressing GABA_A_ receptors in the CM contribute to distinct awareness states. This important discovery implicates a tonic GABA_A_-mediated conductance in the CM that may be relevant for minimally conscious states and other conditions of altered awareness.

## Introduction

The central medial (CM) nucleus forms part of the thalamic rostral intralaminar nuclei (rILN), which also includes the paracentral and central lateral nuclei. The rILN has been implicated in a myriad of processes from arousal and consciousness to action control and executive function (reviewed in Cover and Mathur, 2021). Such functional diversity is perhaps not surprising given the extensive connectivity of the CM with other brain regions. Thus, afferents to the CM span extensive cortical areas comprising somatosensory, motor and orbital frontal cortices, and subcortically the globus pallidus, substantia nigra reticulata, reticular formation, superior colliculus and cerebellum. The CM nucleus in turn projects to anterior cingulate, prelimbic, somatosensory and motor cortices, and subcortically to the striatum and claustrum (Cover and Mathur, 2021). Two main mechanisms have been proposed for how GABA acting *via* GABA_A_ receptors modulates neuronal activity in the brain (reviewed in Chuang and Reddy, 2018). These are either fast-acting GABA_A_ receptors mediating phasic inhibition or slower-acting GABA_A_ receptors mediating tonic inhibition. In the latter case, tonic GABAergic currents are proposed to arise from the binding of GABA to high-affinity GABA_A_ receptors including those expressing the delta subunit (Field et al., 2021; Wyroślak et al., 2023). At least three isoforms of the delta GABA_A_ receptor have been described and, of these, the α4βδ receptor is expressed extrasynaptically in the thalamus (Chuang and Reddy, 2018). By targeting the CM nucleus of the thalamus, Muheyati et al. (2024) revealed a hitherto unappreciated role of delta-expressing GABA_A_ receptors in mediating different levels of consciousness, as inferred in rats by the loss of the righting reflex.

## Methods and findings

**Figure 1.**
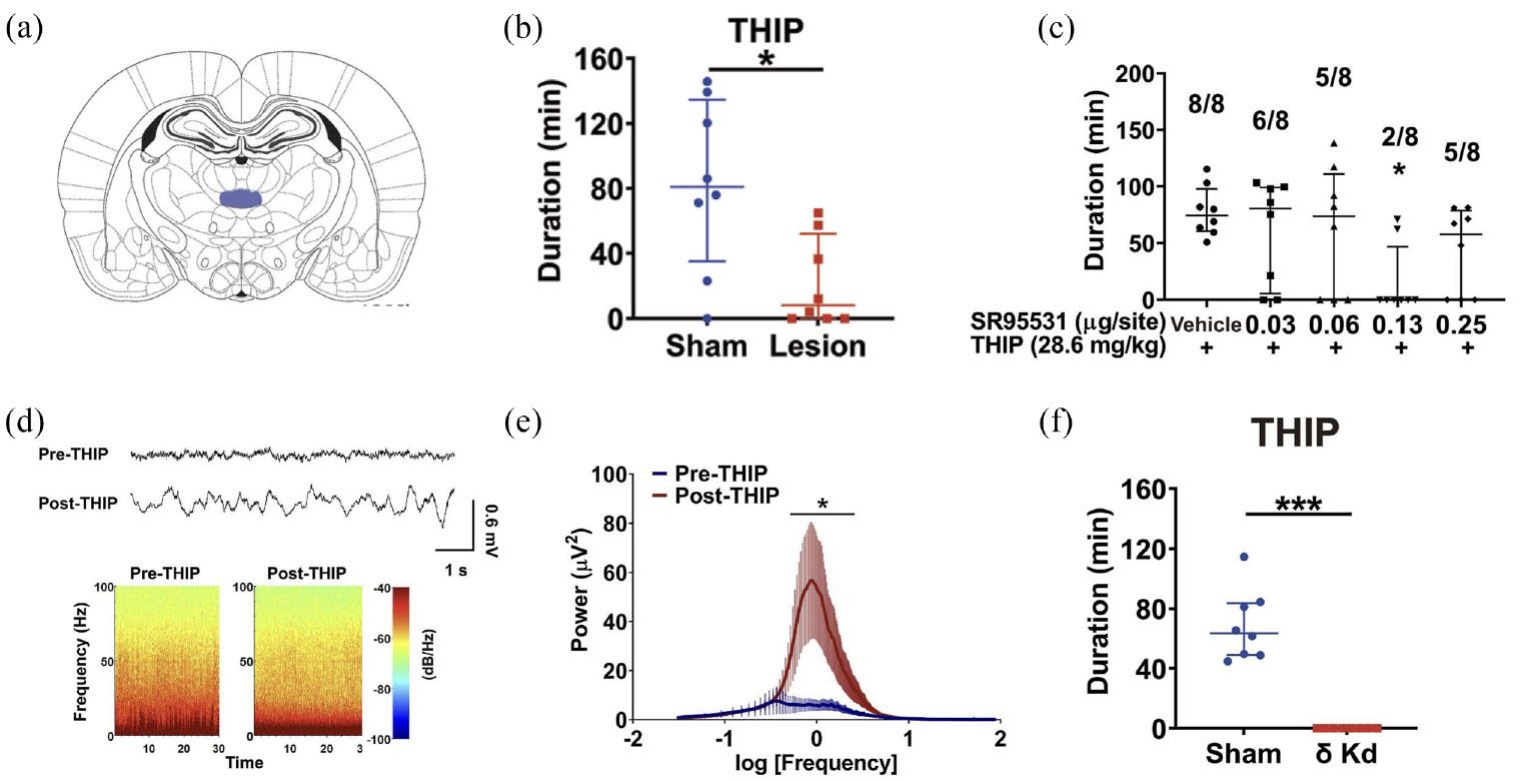
Summary of main findings of Muheyati et al., 2024. (a) Diagram showing the CM in blue (b) CM lesion or (c) infusions of δ GABA A receptor antagonist SR 95531 administered directly into the CM attenuated THIP-induced LORR. (d-e) Delta oscillations were observed in the mPFC following CM THIP infusions. (f) Delta subunit knockdown in the CM resulted in impaired THIP-induced LORR (modified from Muheyati et al., 2024).

Systemic administration of the delta subunit GABA_A_ receptor agonist, 4,5,6,7-tetrahydroisoxazolo [5,4-c] pyridin-3-ol (THIP; also known as Gaboxadol) resulted in a loss of the righting reflex in Sprague Dawley rats, defined by the inability of the rat to right itself within a 1-min period. Confirming the neural locus of this effect, excitotoxic lesions of the CM attenuated the effects of THIP on LORR (Figure 1(b)). Following microinjection of THIP directly into the CM, LORR was induced, an outcome that was attenuated by the GABA_A_ receptor antagonist SR95531 (Figure 1(c)). Of note, intra-CM THIP also increased delta oscillations (0.5–4 Hz) in the medial prefrontal cortex (mPFC) (Figure 1(d) and (e)). Finally, THIP-induced LORR was abolished by viral knockdown of the delta subunit in the CM (Figure 1(f)).

The effects of the GABA_A_ receptor positive allosteric modulator (PAM) diazepam (DZP) were also investigated. Lesions of the CM but not paraventricular (PV) or lateral mediodorsal (MD) nuclei decreased the duration of systemic DZP-induced LORR. However, two unexpected findings were observed. The first was that although CM lesions decreased the duration of LORR when DZP was injected systemically, direct intra-CM infusions of DZP did not result in LORR. The authors argue that this surprising null finding may have arisen by unintended toxicity caused by the high concentration of dimethyl sulfoxide used to dissolve DZP. It was also argued that systemically injected DZP may have affected regions downstream of the CM. A second unexpected finding was that knockdown of delta subunit in the CM attenuated DZP-induced LORR. Since DZP is not thought to modulate delta GABA_A_ receptors, the authors suggest that this unexpected interaction may have arisen in part from increased neuronal excitability in the CM as a consequence of delta subunit depletion.

It should be noted that although LORR has traditionally been used to assess loss of consciousness in animals, there is growing evidence that it is not the most sensitive measure (Gao and Calderon, 2020). Full anaesthetic-induced loss of consciousness implies loss of sensory awareness and pain sensitivity as well as LORR. However, as only the LORR was reported in this study it cannot be inferred if these other processes were also affected. Indeed, mixed findings have previously been noted with respect to the analgesic properties of THIP in rodents (Cheng and Brunner, 1985; Hill et al., 1981).

## Impact

The findings of this article collectively show that delta subunit-expressing GABA_A_ receptors in the CM mediate different states of awareness. The impact of this research arises from the remarkable functional localisation of this receptor to a very specific CM region of the thalamus. These original findings may have relevance for understanding variable consciousness states induced by general anaesthetics or brain trauma. More especially, as discussed below, this research putatively implicates tonic GABA currents in extrasynaptic delta subunit-expressing GABA_A_ receptors in modulating different awareness states.

## Discussion

The functional significance of tonic GABAergic currents in distinct populations of GABAergic interneurons has attracted much recent research interest. For example, Wyroslak and colleagues applied brief N-methyl-D-aspartate (NMDA) receptor stimulation to murine hippocampal slices and measured tonic currents in parvalbumin positive (PV) and somatostatin positive (SST) GABAergic interneurons. Cell type specific changes were observed with PV cells showing a reduction in tonic inhibition mediated by delta-expressing GABA_A_ receptors. In contrast, SST cells showed increased tonic inhibition that depended on alpha5-containing GABA_A_ receptors (Wyroślak et al., 2023). In a recent study, delta GABA_A_ receptors in PV interneurons were selectively silenced using a conditional knockout mouse model and recordings made in slices of the mPFC. THIP administration was found to alter the excitatory/inhibitory ratio in pyramidal neurons leading to a decrease in spontaneous inhibitory postsynaptic currents in wildtype controls but not knockout mice. Increased frequency of spontaneous excitatory postsynaptic currents in the wildtype controls was also observed (Lu et al., 2024).

Beyond neurons, a mechanistic contribution of astrocytes to the regulation of inhibitory tonic currents is also a credible possibility for the reported observations in this study. Astrocytes express the regulatory mechanisms needed for synthesising, releasing and clearing GABA making them key regulators of GABA tone, which underlies the tonic GABA current (Koh et al., 2023). Indeed, astrocytes express GABA_A_ receptors, the primary site of action of anaesthetic drugs such as etomidate and sevoflurane, and mediate crosstalk with neuronal extrasynaptic GABA_A_ receptors to enhance tonic currents (Wang et al., 2024). Other research found that activation of astrocytes in the basal forebrain using designer receptors exclusively activated by designer drugs (DREADDs) facilitated loss of consciousness elicited by isoflurane and also lead to higher delta EEG power compared to controls (Lin et al., 2023).

In their paper Muheyati et al. found that delta oscillations (0.5–4.5 Hz) were enhanced in the rat mPFC following infusions of THIP in the CM nucleus. Although a purely correlative measure, increased delta oscillatory power in this region has previously been linked to changes in consciousness levels. Mechanistically, this effect may be mediated by increased inhibitory tonic currents in the CM nucleus, which in turn serve to entrain delta oscillatory activity in thalamic-cortical pathways. Although delta oscillations have traditionally been associated with sleep and unconsciousness, it has also been shown that they may be responsible for decreasing ATP consumption, thereby replenishing its availability (Dworak et al., 2011). Thus, the co-administration of ketamine-xylazine treatment induced delta oscillations leading to increased levels of ATP and ADP in frontal brain regions (Dworak et al., 2011). Delta oscillations have also been linked to memory weakening during non-rapid eye movement (NREM) sleep (Kim et al., 2019). Moreover, THIP administration was found to increase slow wave activity during wakefulness and rapid eye movement (REM) sleep in mice (Vyazovskiy et al., 2005).

The findings of this study collectively show a necessary and sufficient involvement of the CM nucleus in THIP-induced LORR. However, it is entirely possible that other regions of the rat thalamic rILN, including central lateral and paracentral nuclei also play an important role. For example, previous research found that 50 Hz gamma-frequency stimulation of the central lateral nucleus restored arousal in anaesthetised macaques (Redinbaugh et al., 2020). This included a return of facial and body movement, an opening of the eyes, and increased reactivity to toe-pinch withdrawal. Future research would thus be needed to establish whether delta GABA_A_ receptors in other thalamic rILN nuclei also regulate the LORR in rodents. For example, to validate the localisation of the reported effects, it would be useful to investigate the effects of CM gamma-frequency stimulation on THIP-induced anaesthesia.

In conclusion, research investigating the transitions between wakefulness and anaesthesia-induced unconsciousness is essential to undertake not only to enhance our fundamental understanding of this process but also in the development of potential therapies for disorders of consciousness such as minimally conscious states. Improved insight into the mechanistic actions of anaesthetics will also lead to better outcomes for people following surgery, particularly in older adults who can experience significant adverse side effects from general anaesthesia, including protracted cognitive impairment (Cottrell and Hartung, 2020).
